# Postprandial Inflammation in Obesity: Dietary Determinants, Adipose Tissue Dysfunction and the Gut Microbiome

**DOI:** 10.3390/biom15111516

**Published:** 2025-10-27

**Authors:** Donya Shahamati, Neda S. Akhavan, Sara K. Rosenkranz

**Affiliations:** Department of Kinesiology and Nutrition Sciences, School of Integrated Health Sciences, University of Nevada, Las Vegas, NV 89154, USA; shahamat@unlv.nevada.edu (D.S.);

**Keywords:** obesity, postprandial inflammation, gut-derived endotoxemia, adipose tissue dysfunction, lipopolysaccharides, Toll-like receptor 4, NLRP3 inflammasome, peroxisome proliferator-activated receptors

## Abstract

Obesity is characterized by chronic low-grade inflammation that disrupts metabolic homeostasis and increases cardiometabolic risk. The postprandial period, during which individuals spend much of the day, is a critical window when nutrient absorption, lipid metabolism, and immune activation intersect. In obesity, dysfunctional adipose tissue and impaired gut barrier integrity amplify postprandial inflammatory responses through increased translocation of lipopolysaccharides and altered adipokine secretion. These processes converge on signaling pathways such as Toll-like receptor 4/nuclear factor-κB, c-Jun n-terminal kinase, and the NOD-like receptor family pyrin domain-containing protein 3 (NLRP3) inflammasome, leading to insulin resistance, endothelial dysfunction, and atherogenesis. This review synthesizes evidence on the interplay between gut-derived endotoxemia and adipose tissue dysfunction in postprandial inflammation. We further highlight the modulatory roles of dietary fat quality, plant-based dietary patterns, polyphenols, omega-3 fatty acids, dietary fiber, and nuclear receptor activation, particularly through peroxisome proliferator-activated receptors (PPARs). Emerging evidence indicates that nutritional and pharmacological strategies targeting these mechanisms can attenuate postprandial inflammation and improve metabolic outcomes. A combined approach integrating personalized nutrition, functional foods, and therapies targeting PPAR isoforms may represent a promising avenue for mitigating obesity-associated postprandial inflammation and long-term cardiometabolic complications.

## 1. Introduction

Obesity is one of the most urgent global health challenges we face. The prevalence of obesity among children and adolescents has quadrupled since 1990 [1], and as of 2022, nearly one in six adults (16%) worldwide were living with obesity [2], contributing to a broad range of metabolic disorders, cardiovascular diseases, and certain cancers [3]. Obesity is now considered a chronic disease that is multifaceted, extending beyond the accumulation of adipose tissue, reflecting a complex interplay of genetic, behavioral, environmental, and biological factors [4].

Excess adipose tissue, particularly visceral fat, is metabolically active and acts as a source of pro-inflammatory adipokines, such as tumor necrosis factor-alpha (TNF-α) and interleukin-6 (IL-6), which are associated with systemic insulin resistance and endothelial dysfunction [5]. In addition to bioactive substances originating from adipose tissue, changes in gut barrier integrity, and the translocation of microbial products can be major contributors to chronic low-grade inflammation [6]. The postprandial state, which is the period following food intake, is a critical window for investigating inflammatory processes. Most people spend a substantial portion of the day in this state [7], when the body is repeatedly exposed to metabolic and immune challenges from meals, including increases in blood glucose, triglycerides, and endotoxins that stimulate inflammatory responses. This is particularly important in the context of the typical American diet, which is often characterized by frequent eating occasions and meals rich in saturated fats, refined carbohydrates, and added sugars—dietary patterns that are known to amplify postprandial inflammatory responses. Postprandial inflammation is often exacerbated due to both adipose tissue dysfunction and gut-derived signals such as endotoxemia within populations with obesity [8]. This review provides the systemic and gut-mediated mechanisms underlying postprandial inflammation, clarifies their interactions, and evaluates dietary interventions designed to mitigate these inflammatory responses and their cardiometabolic consequences.

## 2. Inflammation: From Acute Protection to Chronic Meta-Inflammation

Inflammation is a biological response that protects against harmful agents, including pathogens, toxins, and tissue damage [9]. This process is essential for the survival of multicellular organisms, maintaining health by promoting healing and defending against harmful agents [10]. The cardinal signs of inflammation include pain, redness, heat, swelling, and loss of function; where these acute responses are typically short-lived, acting to resolve the injury or infection and facilitate tissue repair [11,12].

While inflammation serves critical protective functions, it can become harmful when chronic or dysregulated. Mechanistically, the adaptive immune system produces cytokines and chemokines that perpetuate inflammation and contribute to tissue damage [13]. Inflammation associated with excess adiposity is often referred to as “meta-inflammation”, a prolonged inflammatory response that differs significantly from classical inflammation [14,15]. Unlike acute inflammation, which is localized and resolves after the harmful agent is neutralized, meta-inflammation persists, is often low-grade, and is associated with metabolic disturbances and comorbid conditions, such as insulin resistance, cardiovascular diseases, and Alzheimer’s disease [16,17].

## 3. Inducers, Sensors, and Signaling Pathways in Obesity-Related Inflammation

In the context of obesity, a broad range of exogenous and endogenous factors can initiate inflammation. Exogenous inducers include microbial products such as pathogen-associated molecular patterns (PAMPs), among which, bacterial lipopolysaccharides (LPS) are particularly important. These molecules can activate Toll-like receptors (TLRs) on immune cells, initiating pro-inflammatory signaling cascades [18]. In addition to microbial products, non-microbial dietary components such as saturated fatty acids, processed sugars, and artificial food additives induce inflammation, further exacerbating metabolic stress in obesity [19]. Endogenous inducers of inflammation are equally important and often reflect an altered metabolic and tissue environment associated with excess adiposity. Cytokines such as TNF-α and IL-6, which are primarily produced by activated immune cells, contribute to systemic inflammation [20]. Damage-associated molecular patterns (DAMPs) that result from tissue stress, hypoxia, or necrosis also function as inflammatory signals, engaging innate immune receptors and perpetuating low-grade inflammation [21]. Plasma-derived proteins, such as C-reactive protein (CRP), are not only biomarkers, but also play an active role in inflammatory processes [22]. Meanwhile, extracellular matrix fragments produced during tissue remodeling provide persistent danger signals that sustain inflammatory activation [23].

**Figure 1:**
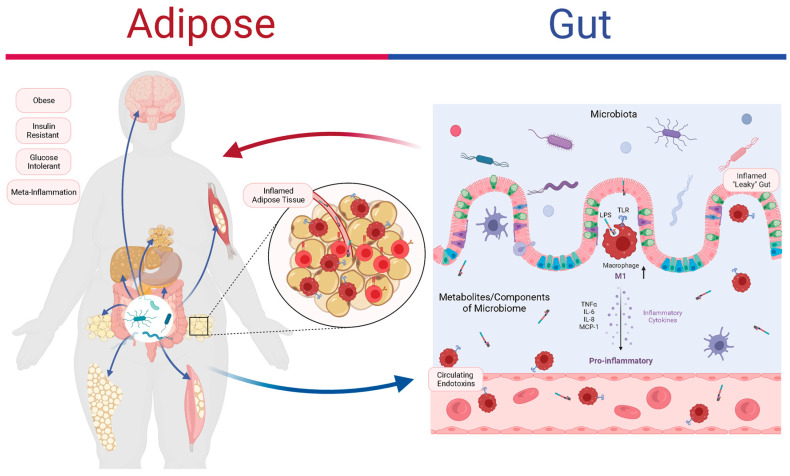
Conceptual model of gut–adipose–immune interactions contributing to metabolic inflammation. Reprinted with permission from Ref. [28], under the terms of the Creative Commons Attribution (CC BY) license. LPS, lipopolysaccharides; TLR, Toll-like receptors; TNF-α, tumor necrosis factor alpha; IL-6, interleukin-6; IL-8, interleukin-8; MCP-1, monocyte chemoattractant protein-1.

At the cellular level, several pattern recognition receptors and signaling pathways mediate the effects of these inducers in the context of obesity. These can be classified as ‘nutrient-sensing danger signals’, providing insight into their roles in inflammation. The Toll-like receptor 4/Nuclear Factor kappa B (TLR4/NF-κB) axis, activated by LPS and saturated fatty acids, induces many pro-inflammatory genes [24]. The c-Jun n-terminal kinase (JNK) cascade also promotes inflammation and interferes with insulin receptor signaling, linking immune activation to metabolic dysfunction. In parallel, pro-inflammatory signals can impair the phosphoinositide 3-kinase (PI3K)/Akt pathway, a key downstream mediator of insulin receptor substrates (IRS-1/IRS-2), reducing glucose uptake and further disrupting metabolic homeostasis [16]. Specifically, activated JNK phosphorylates insulin receptor substrates (IRS-1/IRS-2) on serine residues (e.g., Ser-307), impairing their tyrosine phosphorylation and thus disrupting insulin signaling in obesity and insulin resistance models [25]. The NOD-like receptor family pyrin domain-containing protein 3 (NLRP3) inflammasome integrates metabolic stress, triggered by lipotoxicity, oxidative stress, and cellular debris. Its activation leads to the release of pro-inflammatory cytokines, such as interleukin-1β (IL-1β) and interleukin-18 (IL-18), which reinforce obesity-related inflammation and metabolic impairment [26]. Notably, multiple inflammatory pathways—including TLR4/NF-κB, JNK, and NLRP3—converge on central cellular hubs such as the endoplasmic reticulum (ER) and mitochondria. Stress at these hubs disrupts protein folding and energy production, worsening metabolic dysfunction, which highlights their potential as targets for therapeutic intervention. By targeting these convergence points, therapies might simultaneously act on several signaling pathways, presenting a promising approach to mitigating inflammation in obesity [27]. As illustrated in Figure 1 [28], gut dysbiosis and impaired intestinal barrier integrity promote systemic endotoxemia and immune activation, which in turn, drive macrophage infiltration and adipose tissue inflammation. 

## 4. Adipose Tissue Dysfunction in Obesity

### 4.1. Macrophage Recruitment and Phenotypic Shift

With the progression of obesity, adipose tissue undergoes extensive remodeling characterized by adipocyte hypertrophy, local hypoxia, and a marked increase in immune cells infiltrating the adipose tissue, where they release inflammatory signals that affect adipocyte function. One of the hallmarks of this remodeling process is the accumulation of macrophages within the adipose tissue [29,30,31]. In lean states, adipose tissue macrophages are predominantly the anti-inflammatory M2 phenotype, which helps maintain tissue homeostasis. However, in obesity, these macrophages undergo a phenotypic shift toward the pro-inflammatory M1 state [32]. This transition results in increased secretion of cytokines such as TNF-α, IL-6, and monocyte chemoattractant protein-1 (MCP-1), which collectively drive systemic meta-inflammation and can contribute to the development of insulin resistance [33].

### 4.2. Adipokine Dysregulation

Adipose tissue dysfunction in obesity is also characterized by significant dysregulation of adipokine secretion. Among the pro-inflammatory adipokines, leptin is typically elevated in obesity and has important immunomodulatory functions. Through the activation of signaling pathways such as Janus kinase 2/Signal transducer and activator of transcription 3 (JAK2/STAT3), Mitogen-activated protein kinase (MAPK), and Phosphoinositide 3-kinase (PI3K), leptin promotes the production of IL-6 and TNF-α, contributing to oxidative stress, even in the context of leptin resistance [34,35]. Resistin, another pro-inflammatory adipokine, is primarily secreted by macrophages and has been shown to activate the NF-κB pathway through binding to its receptor Adenylyl Cyclase-Associated Protein 1 (CAP1), thereby exacerbating insulin resistance and promoting endothelial dysfunction characterized by reduced nitric oxide bioavailability and increased production of vasoconstrictive agents [36]. In contrast, adiponectin is considered an anti-inflammatory adipokine that is significantly reduced in obesity. Adiponectin typically inhibits NF-κB signaling and cytokine expression, while improving insulin sensitivity; its decrease in obesity further amplifies inflammatory and metabolic imbalances [37].

### 4.3. Adipocyte Hypertrophy and Endocrine Dysregulation

Adipocyte hypertrophy plays a central role in promoting the inflammatory environment in obesity. As adipocytes enlarge, their production of protective adipokines, such as adiponectin, decreases, while the release of pro-inflammatory mediators, including TNF-α and IL-6, increases [14,38,39]. The resulting imbalance disrupts normal endocrine signaling, contributing to systemic insulin resistance and metabolic dysfunction. 

Additionally, due to insufficient angiogenesis, hypertrophic adipose tissue often becomes hypoxic [37,40], which accelerates adipocyte apoptosis and necrosis, thereby exacerbating tissue dysfunction [39]. Cellular breakdown products released from dying adipocytes recruit immune cells, particularly macrophages, into adipose tissue. The inflammatory response is therefore amplified, promoting a cycle of cellular stress and immune activation. 

In addition to cytokine dysregulation and hypoxia, hypertrophic adipocytes promote the release of free fatty acids (FFAs). These FFAs act as endogenous ligands for TLRs, particularly TLR4, on macrophages and other immune cells, initiating pro-inflammatory signaling cascades [41]. Stress signals associated with FFAs also activate inflammasome complexes, such as the NLRP3 inflammasome, which further drives the production of inflammatory mediators, including IL-1β and IL-18. This process not only perpetuates local adipose tissue inflammation but also low-grade systemic inflammation, contributing to the development of obesity-related metabolic diseases [39,42].

## 5. Dietary Fat Composition and Inflammatory Effects

### 5.1. Chronic Effects

Dietary fat composition plays a significant role in inflammation. While chronic saturated fatty acid (SFA) intake is often pro-inflammatory, Omega-3 polyunsaturated fatty acids (n-3 PUFA) and monounsaturated fatty acids (MUFA) generally exert anti-inflammatory effects and improve insulin sensitivity [43,44]. The role of omega-6 (n-6 PUFA) is more nuanced: although some metabolites can promote inflammation, linoleic acid—the predominant n-6 PUFA in the diet—does not appear to be strongly pro-inflammatory, highlighting a more complex relationship than previously understood [45]. Saturated fatty acids are known to stimulate TLR4 pathways, thereby promoting inflammation [46]. In contrast, n-3 PUFA and MUFA operate through different mechanisms, such as upregulating Peroxisome Proliferator-Activated Receptor gamma (PPARγ) activity, which contributes to the downregulation of inflammatory markers and improvement in insulin sensitivity [47,48]. In a mouse model of diet-induced obesity, the animals were initially fed a high-fat (HF) diet for 8 weeks to induce obesity. They were then randomly assigned to continue the HF diet or switch to a fat-substituted diet, in which saturated fats were partially replaced with unsaturated fatty acids at 10%, 20%, or 30% of total fat, for an additional 8 weeks. Study results showed that replacing saturated fats with unsaturated fatty acids reduced hypothalamic inflammation, improved insulin sensitivity, and decreased adiposity in an animal model of obesity [49]. Large cohort studies also concur with these findings, indicating that higher intake of MUFAs and n-3 PUFAs, in place of saturated fats, is associated with improved insulin sensitivity, reduced systemic inflammation, and lower cardiometabolic risk [50,51].

### 5.2. Acute Postprandial Effects

The postprandial period is when active nutrient absorption and metabolic processing predominate [52]. Consumption of a high-fat meal, such as those typical of fast food, can significantly increase circulating triglyceride (TG) concentrations during this period [53]. In individuals with obesity or impaired lipid metabolism, elevated TG levels may persist for several hours, thereby extending the postprandial state [54]. Prolonged exposure to elevated TG is associated with increased oxidative stress and activation of inflammatory pathways, including enhanced macrophage recruitment, which intensifies systemic inflammation [55].

High-fat meals increase intestinal permeability and facilitate chylomicron-mediated transport of LPS into the circulation, resulting in metabolic endotoxemia [56,57]. Elevated postprandial plasma LPS activates TLR4 signaling in immune cells and adipose tissue, stimulating the production of pro-inflammatory cytokines such as TNF-α and IL-6 [57,58]. Similarly, dietary phenolic compounds, such as those in high-phenol virgin olive oil, exhibit anti-inflammatory effects by reducing postprandial plasma LPS and altering gene expression in peripheral blood mononuclear cells, likely through suppression of NF-κB, activator protein 1 (AP-1), and MAPK pathways [58,59]. The compositions of the test meal or food, specifically the types of fats and carbohydrates, are primary determinants of postprandial inflammatory responses. In a randomized crossover controlled-feeding trial, single meals high in saturated fats were shown to increase postprandial inflammatory markers, such as high-senstivity CRP (hs-CRP) and erythrocyte sedimentation rate (ESR). In contrast, meals rich in monounsaturated fats and fiber did not trigger such responses [60]. Similarly, saturated-fat-rich meals induced elevated postprandial expression of inflammatory genes, such as IL-1β, a response that was mitigated by unsaturated-fat- and fiber-rich meals [61]. At the molecular level, enzymes such as Stearoyl-CoA Desaturase 1 (SCD1), which mediate the production of MUFAs from SFAs, are implicated in modulating obesity-related inflammation [46].

**Figure 2:**
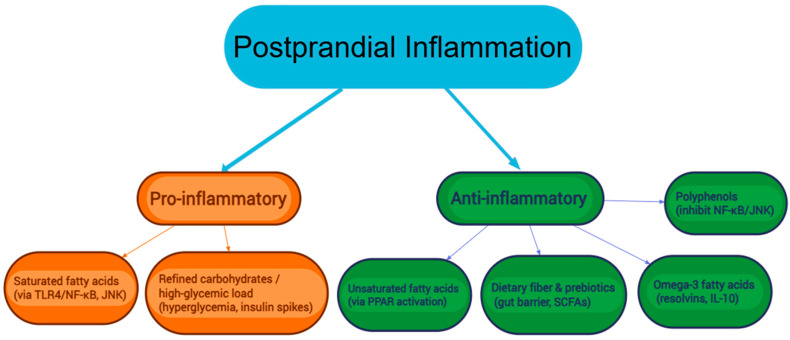
Proposed dietary modulators of postprandial inflammation. TLR4, Toll-like receptor 4; NF-κB, Nuclear factor kappa-light-chain-enhancer of activated B cells, JNK, c-Jun N-terminal kinase; PPAR, Peroxisome proliferator-activated receptor, SCFAs Short-chain fatty acids; IL-10; Interleukin-10.

Diets high in saturated fats, characteristic of Western dietary patterns, increase the production of pro-inflammatory cytokines. Similarly, highly processed and high-glycemic carbohydrates increase postprandial glucose and insulin levels, thereby promoting inflammation. Conversely, meals rich in unsaturated fats, such as those typical of the Mediterranean diet, attenuate postprandial pro-inflammatory responses, partly by limiting NF-κB activation [62]. An overview of key dietary factors driving pro- and anti-inflammatory postprandial pathways is illustrated in Figure 2.

## 6. Postprandial Inflammation: From Acute Responses to Chronic Diseases

Research indicates that obesity exacerbates postprandial inflammatory responses, particularly after consuming high-fat and high-glycemic load meals. As compared with individuals with normal weight, people with obesity exhibit higher postprandial glucose, insulin, TG, and inflammatory markers such as IL-6, TNF-α, and CRP [63,64,65]. These acute increases in cytokines are associated with endothelial activation, oxidative stress, and postprandial lipemia, collectively contributing to a pro-coagulant state and long-term cardiometabolic risk, including atherosclerosis and metabolic syndrome [66,67,68]. Additionally, the magnitude of postprandial TG and IL-6 responses is more pronounced in individuals with obesity, particularly following larger caloric loads, promoting systemic inflammation and cardiovascular complications [69]. High levels of postprandial remnant and TG-rich lipoproteins can infiltrate the endothelium, activate macrophages, and prolong oxidative stress, reinforcing the link between postprandial inflammation and atherogenesis [67]. Accumulation of these TG-rich remnants can further activate leukocytes and endothelial cells, upregulate adhesion molecules, and stimulate foam cell formation, promoting endothelial dysfunction and insulin resistance that underlie metabolic diseases such as type 2 diabetes and atherosclerosis [70]. As illustrated in Figure 1, repeated exposure to these acute postprandial inflammatory events can progressively transition into chronic low-grade inflammation, establishing a mechanistic continuum from meal-induced responses to long-term metabolic and cardiovascular diseases.

## 7. Nuclear Receptor Regulation (PPARs)

Peroxisome proliferator-activated receptors (PPARs)—including PPAR alpha (PPARα), PPAR beta/delta (PPARβ/δ), and PPARγ—are ligand-activated nuclear receptors that coordinate lipid metabolism, glucose homeostasis, and inflammation [71]. In an obese state, chronic overnutrition increases circulating FFAs and TG-rich lipoproteins, which generally act as endogenous ligands for the activation of PPAR. However, obesity often disrupts PPAR signaling in adipose tissue, liver, and vascular cells, impairing fatty acid oxidation and promoting inflammatory pathways [71,72].

The reduced expression or activity of PPARα and PPARβ/δ in obesity contributes to impaired postprandial lipid clearance, prolonging the elevation of TG-rich lipoproteins after meals. This delayed clearance promotes endothelial activation, oxidative stress, and low-grade inflammation, amplifying postprandial increases in IL-6, CRP, and TNF-α [71,73]. PPARγ is abundant in adipose tissue and macrophages, promoting insulin sensitivity, M2 macrophage polarization, and adiponectin secretion, while suppressing NF–κB–mediated cytokine expression [48]. Pharmacological activation of PPARγ through agonists such as thiazolidinediones (TZDs), mitigates large increases in postprandial inflammatory cytokine production and improves insulin signaling [74]. Moreover, dual PPARα/γ agonists provide synergistic metabolic benefits by enhancing hepatic and adipose lipid metabolism, restoring gut–adipose communication, reducing endotoxemia, and diminishing macrophage activation, thereby lowering both fasting and postprandial inflammation [75].

Beyond PPARs, other nutrient-sensitive nuclear receptors and kinases play complementary roles in regulating postprandial inflammation and metabolic homeostasis. The farnesoid X receptor (FXR), activated by bile acids, modulates lipid and glucose metabolism through the FXR-Fibroblast growth factor 19 (FGF19) axis and suppresses intestinal endotoxin absorption, thereby limiting inflammation after high-fat meals [76]. Similarly, the liver X receptor (LXR) acts as a cholesterol sensor that promotes reverse cholesterol transport and induces anti-inflammatory gene expression in macrophages, reducing cytokine production and lipid accumulation in vascular tissue [77]. In parallel, AMP-activated protein kinase (AMPK) functions as a central energy sensor that integrates nutrient status with inflammatory signaling. Activation of AMPK by fasting, exercise, or intake of polyphenols enhances fatty acid oxidation and inhibits NF-κB and JNK pathways, thereby counteracting the pro-inflammatory effects of nutrient overload [78,79].

## 8. Nutritional Strategies for Mitigating Postprandial and Chronic Inflammation in Obesity

### 8.1. Dietary Patterns

Adherence to plant-based, Mediterranean, or DASH dietary patterns, particularly those rich in unsaturated fats such as extra-virgin olive oil and nuts, is consistently associated with reduced systemic inflammation and improved endothelial function. A recent meta-analysis demonstrated that the Mediterranean dietary pattern enriched with olive oil significantly improved inflammatory markers and endothelial function compared to low-fat diets [80]. However, variability in the dosage of olive oil and nuts across different trials introduces uncertainty in dose–response outcomes. Mechanistic reviews indicate that these dietary patterns influence immune responses and gut microbiota composition, thereby reducing inflammation [81]. Experimental studies have shown that substituting saturated fats with olive oil and nuts in Western-style diets reduces atherosclerosis, foam cell formation, and inflammatory cytokine expression [82,83]. In one study, male low-density lipoprotein receptor knockout (Ldlr–/–) mice were fed a Western diet or a diet enriched with extra-virgin olive oil and nuts for 3 months. The diet enriched with extra-virgin olive oil and nuts improved plasma lipid profiles and reduced vascular inflammation in these atheroprone mice, which are genetically predisposed to developing atherosclerosis [83].

Plant-based diets also offer potent anti-inflammatory benefits. Intervention studies demonstrate significant reductions in obesity-related inflammatory profiles with plant-based dietary patterns [84]. Diets rich in fruits, vegetables, and plant oils—sources of phytochemicals and antioxidants—produce significant decreases in CRP among people with overweight, obesity, and diabetes [85]. Cross-sectional data further indicate that higher intake of healthy plant foods (whole grains, fruits, vegetables, unsaturated fats) is associated with lower hs-CRP and Transforming Growth Factor-beta (TGF-β) levels [86]. Comprehensive reviews support the notion that plant-based diets can help mitigate chronic metabolic inflammation, leading to improvements in bodyweight, lipid profiles, and inflammatory biomarkers [87].

### 8.2. Functional Foods and Bioactive Compounds

Beyond basic nutrient composition, dietary components including polyphenols, n-3 PUFAs, and dietary fibers modulate inflammatory processes associated with obesity, affecting both chronic low-grade inflammation and postprandial inflammatory spikes [88,89]. These bioactive foods act through several complementary mechanisms, including the inhibition of NF-κB and JNK signaling, the activation of AMP-activated protein kinase (AMPK) and PPARγ pathways, and the regulation of G-protein-coupled receptors such as GPR120.

#### 8.2.1. Polyphenols

Polyphenol-rich foods, such as berries, green tea, and cocoa, suppress adipose tissue inflammation by downregulating NF-κB and JNK signaling and reducing secretion of TNF-α, IL-6, and MCP-1. Many of these effects are partially PPARγ-dependent, promoting M2 macrophage polarization and increased adiponectin expression in adipose tissue [89,90].

In a randomized controlled crossover study of adults with impaired fasting glucose and type 2 diabetes [91], adding extra-virgin olive oil—rich in hydroxytyrosol and other phenolic compounds—to a standardized meal significantly reduced postprandial plasma lipopolysaccharide (LPS) and zonulin concentrations compared with a control meal without olive oil. Mechanistically, olive-oil polyphenols enhance the expression of tight-junction proteins, including occludin and claudin-1, and suppress activation of the LPS–TLR4–NF-κB pathway, thereby attenuating downstream release of IL-6 and TNF-α.

Although some studies using isolated polyphenol supplements have shown limited effects in the acute postprandial period, sustained intake of polyphenol-rich foods has demonstrated consistent benefits in reducing obesity-related inflammatory responses [92,93]. Wang et al. reviewed human and preclinical data and reported that dietary polyphenols are biotransformed by the gut microbiota into phenolic metabolites that increase short-chain fatty acid (SCFA) production, strengthen epithelial tight junctions, and inhibit NF-κB and MAPK signaling in intestinal and adipose tissues [28]. Similarly, Mao et al. systematically analyzed randomized controlled trials and showed that polyphenol interventions—particularly from olive oil, tea, and berries—significantly reduced circulating CRP, IL-6, and TNF-α in individuals with obesity or metabolic syndrome [94].

#### 8.2.2. Omega-3 Fatty Acids

Marine n-3 PUFAs, including eicosapentaenoic acid (EPA) and docosahexaenoic acid (DHA), increase pro-resolving lipid mediators such as Resolvin D1 and anti-inflammatory cytokines like interleukin-10, while reducing IL-6 and MCP-1. These effects support their role in both chronic inflammation and postprandial resolution pathways [95,96].

In a randomized, dose–response trial, Skulas-Ray et al. demonstrated that supplementation with EPA+DHA for several weeks led to a dose-dependent reduction in both fasting and postprandial triglyceride concentrations. Specifically, participants receiving a higher therapeutic dose (~3.4 g/d) exhibited significantly lower postprandial TG levels compared with those on a lower dose or placebo [97].

In addition to their lipid-lowering effects, n-3 PUFAs may also attenuate postprandial endotoxemia. Lyte et al. compared the postprandial responses to meals differing in fatty acid composition and found that those enriched with long-chain n-3 PUFAs resulted in lower circulating endotoxin (LPS) levels compared with meals rich in saturated or n-6 PUFAs [98].

#### 8.2.3. Dietary Fibers and Prebiotics

Dietary fibers and prebiotics improve gut barrier function, reduce macrophage infiltration, and downregulate adipose tissue inflammation, collectively improving insulin sensitivity [43,99]. Acute meal studies show that the type and amount of fiber can markedly influence postprandial responses. In a randomized crossover trial in women with overweight, meals enriched with higher levels of β-glucan (up to 3.7 g) and resistant starch (up to 6.5 g) significantly lowered postprandial glucose and insulin peaks compared with low-fiber meals [100]. 

Longer-term interventions with prebiotic fibers reinforce these effects through gut-level mechanisms. In the *Food4Gut* randomized placebo-controlled trial, adults with obesity consumed inulin-type fructans (16 g/day) for three months, or a maltodextrin placebo alongside standard dietary advice. The inulin group showed a reduction in fecal calprotectin, a marker of intestinal inflammation, along with increased abundance of *Bifidobacterium*, a key genus linked to improved gut barrier integrity and anti-inflammatory effects. Although total fecal SCFAs were not significantly altered, shifts in other microbial metabolites indicated enhanced colonic fermentation and potential modulation of gut-derived immune signaling [101]. Similarly, in a 12-week open-label randomized controlled pilot study in individuals with metabolic syndrome, supplementation with a prebiotic fiber blend combined with a healthy diet resulted in a decrease in hs-CRP from baseline compared with diet advice alone [102].

Emerging evidence indicates that gut microbiota-derived metabolites, including SCFAs, secondary bile acids, and tryptophan metabolites, play a crucial role in linking intestinal activity with adipose tissue inflammation. SCFAs such as acetate, propionate, and butyrate regulate adipose and systemic inflammation through G-protein-coupled receptors (GPR41 and GPR43) and AMP-activated protein kinase (AMPK) signaling, which enhance adiponectin secretion and suppress NF-κB activation [103]. Bile acids also participate in this regulatory network by activating FXR and Takeda G protein–coupled receptor 5 (TGR5), thereby improving lipid metabolism, promoting energy expenditure, and reducing macrophage infiltration in adipose tissue [76]. Similarly, microbiota-derived tryptophan metabolites, such as indole-3-propionic acid and indole-3-aldehyde—activate the aryl hydrocarbon receptor (AhR), which helps maintain intestinal barrier integrity and attenuates inflammation in adipose tissue [104,105].

### 8.3. Synergistic Approaches

Synergistic dietary approaches that combine n-3 PUFAs, polyphenol-rich plant foods, fermented foods, and prebiotic fibers involve multiple complementary metabolic and inflammatory pathways. Omega-3 fatty acids exert anti-inflammatory effects by suppressing NF-κB and JNK signaling pathways while simultaneously activating PPARγ, which improves insulin sensitivity and lipid metabolism [90]. Polyphenols, which are abundant in fruits, vegetables, tea, and cocoa, further enhance endothelial function, attenuate oxidative stress and inflammation via the mitogen-activated protein kinase (MAPK) pathways [106]. Fermented foods contain probiotics and bioactive peptides that improve gut barrier integrity and reduce endotoxemia, thereby limiting systemic inflammation [104]. Prebiotic fibers, such as inulin and fructooligosaccharides, selectively promote the growth of beneficial gut microbiota, increase the production of SCFAs, and enhance intestinal barrier function [103]. Together, these interventions act synergistically to reduce adipose tissue inflammation, improve postprandial metabolic responses, and ultimately lower the risk of obesity-related cardiometabolic disorders [88,90,103,104]. Key risk and protective factors are summarized in Table 1.

## 9. Conclusions

Postprandial inflammation signifies the connection between obesity and chronic cardiovascular and metabolic diseases. This inflammation arises from the interaction between gut-derived endotoxemia and adipose tissue dysfunction, converging on pathways such as TLR4/NF-κB, JNK, and the NLRP3 inflammasome. The translocation of gut endotoxins and inflammation driven by adipose tissue exacerbate inflammatory responses after high-fat meals in individuals with obesity. Notably, excess adipose tissue, characterized by hypertrophy, endocrine dysregulation, and immune cell infiltration, sustains low-grade chronic inflammation, further advancing insulin resistance, endothelial dysfunction, and atherogenesis. At its core, the gut–adipose axis drives disease by perpetuating a relentless cycle of inflammation and metabolic disruption.

Dietary fat quality, gut barrier integrity and function, and nuclear receptor activity—particularly PPARs—are important modulators of postprandial inflammation. Diets enriched in unsaturated fatty acids, polyphenols, and dietary fibers can attenuate postprandial endotoxemia, suppress pro-inflammatory cytokine production, and improve metabolic outcomes. Pharmacological and/or nutritional strategies targeting PPAR activation may hold promise for restoring lipid metabolism, promoting anti-inflammatory signaling, and reducing excessive postprandial inflammatory responses. A strategy that combines personalized nutrition, functional foods, and targeted PPAR modulation may be necessary to disrupt the cycle of postprandial inflammation and long-term metabolic and cardiovascular complications in obesity. Future research should prioritize interventions that leverage the combined effects of dietary modification, gut microbiota modulation, and nuclear receptor activation to reduce obesity-related postprandial inflammation and associated health outcomes.

## Figures and Tables

**Table 1:** Risk and protective factors influencing postprandial inflammation in obesity.

Risk Factors	Key References	Protective Factors	Key References
Obesity → Chronic low-grade meta-inflammation	[4,14,27]	Dietary patterns → Mediterranean, plant-based, DASH diets lower systemic and postprandial inflammation	[80,81,82,84]
Gut-derived endotoxemia → High-fat meals increase gut permeability and LPS translocation	[6,56,57]	Functional foods → Polyphenols, omega-3 fatty acids, dietary fiber, fermented foods reduce endotoxemia and promote anti-inflammatory pathways	[89,91,97,99,104]
Saturated fats → Stimulate inflammatory signaling, worsen postprandial endotoxemia	[46,60,61]	Fat quality → MUFA and omega-3 fatty acids attenuate inflammatory pathways (via PPAR activation)	[44,47,48,49]
Adipose tissue dysfunction → Hypertrophy, hypoxia, immune cell infiltration → ↑TNF-α, ↑IL-6, ↓adiponectin	[29,32,37,39]	Synergistic approaches → Combining unsaturated fats, polyphenols, pre/probiotics, and omega-3s enhances metabolic and immune benefits	[88,90,103,104]

Summary of key risk and protective factors influencing postprandial inflammation. In this table, **→** denotes a mechanistic relationship where one factor promotes or leads to the observed outcome; **↑** indicates an increase or upregulation; **↓** indicates a decrease or downregulation in specific biomarkers or metabolic responses.

## Data Availability

No new data were created or analyzed in this study.

## References

[1] World Health Organization (2024). One in Eight People Are Now Living with Obesity [Internet].

[2] World Obesity Federation (2022). Prevalence of Obesity [Internet].

[3] World Health Organization (2021). Obesity and Overweight [Internet].

[4] Gregor M.F., Hotamisligil G.S. (2011). Inflammatory mechanisms in obesity. Annu. Rev. Immunol..

[5] Saltiel A.R., Olefsky J.M. (2017). Inflammatory mechanisms linking obesity and metabolic disease. J. Clin. Investig..

[6] Cani P.D., Amar J., Iglesias M.A., Poggi M., Knauf C., Bastelica D., Neyrinck A.M., Fava F., Tuohy K.M., Chabo C. (2007). Metabolic endotoxemia initiates obesity and insulin resistance. Diabetes.

[7] El Khoury D., Hwalla N., Frochot V., Lacorte J.M., Chabert M., Kalopissis A.D. (2010). Postprandial metabolic and hormonal responses of obese dyslipidemic subjects with metabolic syndrome to test meals, rich in carbohydrate, fat or protein. Atherosclerosis.

[8] Mazidi M., Valdes A.M., Ordovas J.M., Hall W.L., Pujol J.C., Wolf J., Hadjigeorgiou G., Segata N., Sattar N., Koivula R. (2021). Meal-induced inflammation: Postprandial insights from the Personalised REsponses to DIetary Composition Trial (PREDICT) study in 1000 participants. Am. J. Clin. Nutr..

[9] Chen L., Deng H., Cui H., Fang J., Zuo Z., Deng J., Li Y., Wang X., Zhao L. (2017). Inflammatory responses and inflammation-associated diseases in organs. Oncotarget.

[10] Medzhitov R. (2010). Inflammation 2010: New adventures of an old flame. Cell.

[11] Medzhitov R. (2008). Origin and physiological roles of inflammation. Nature.

[12] Hannoodee S., Nasuruddin D.N. (2024). Acute Inflammatory Response.

[13] Balkwill F., Mantovani A. (2001). Inflammation and cancer: Back to Virchow?. Lancet.

[14] Hotamisligil G.S. (2006). Inflammation and metabolic disorders. Nature.

[15] Baker S.R., Hayden M.S., Ghosh S. (2011). NF-κB, inflammation, and metabolic disease. Cell.

[16] Hotamisligil G.S. (2017). Inflammation, metaflammation and immunometabolic disorders. Nature.

[17] Kandimalla R., Thirumala V., Reddy P.H. (2017). Is Alzheimer’s disease a type 3 diabetes? A critical appraisal. Biochim. Biophys. Acta BBA-Mol. Basis Dis..

[18] Baothman O.A., Zamzami M.A., Taher I., Abubaker J., Abu-Farha M. (2016). The role of gut microbiota in the development of obesity and diabetes. Lipids Health Dis..

[19] Tristan Asensi M., Napoletano A., Sofi F., Dinu M. (2023). Low-grade inflammation and ultra-processed foods consumption: A review. Nutrients.

[20] Shoelson S.E., Lee J., Goldfine A.B. (2006). Inflammation and insulin resistance. J. Clin. Investig..

[21] Roh J.S., Sohn D.H. (2018). Damage-associated molecular patterns in inflammatory diseases. Immune Netw..

[22] Bistrian B.R. (1999). Acute phase proteins and the systemic inflammatory response. Crit. Care Med..

[23] Sorokin L. (2010). The impact of the extracellular matrix on inflammation. Nat. Rev. Immunol..

[24] Cavalcante-Silva L.H., Galvão J.G., Silva J.S., Sales-Neto J.M., Rodrigues-Mascarenhas S. (2015). Obesity-driven gut microbiota inflammatory pathways to metabolic syndrome. Front. Physiol..

[25] Feng J., Lu S., Ou B., Liu Q., Dai J., Ji C., Zhou H., Huang H., Ma Y. (2020). The role of JNk signaling pathway in obesity-driven insulin resistance. Diabetes Metab. Syndr. Obes. Targets Ther..

[26] Zhang N.P., Liu X.J., Xie L., Shen X.Z., Wu J. (2019). Impaired mitophagy triggers NLRP3 inflammasome activation during the progression from nonalcoholic fatty liver to nonalcoholic steatohepatitis. Lab. Investig..

[27] Zatterale F., Longo M., Naderi J., Raciti G.A., Desiderio A., Miele C., Beguinot F. (2020). Chronic adipose tissue inflammation linking obesity to insulin resistance and type 2 diabetes. Front. Physiol..

[28] Wang C., Yi Z., Jiao Y., Shen Z., Yang F., Zhu S. (2023). Gut microbiota and adipose tissue microenvironment interactions in obesity. Metabolites.

[29] Weisberg S.P., McCann D., Desai M., Rosenbaum M., Leibel R.L., Ferrante A.W. (2003). Obesity is associated with macrophage accumulation in adipose tissue. J. Clin. Investig..

[30] Xu H., Barnes G.T., Yang Q., Tan G., Yang D., Chou C.J., Sole J., Nichols A., Ross J.S., Tartaglia L.A. (2003). Chronic inflammation in fat plays a crucial role in the development of obesity-related insulin resistance. J. Clin. Investig..

[31] Kanda H., Tateya S., Tamori Y., Kotani K., Hiasa K.I., Kitazawa R., Kitazawa S., Miyachi H., Maeda S., Egashira K. (2006). MCP-1 contributes to macrophage infiltration into adipose tissue, insulin resistance, and hepatic steatosis in obesity. J. Clin. Investig..

[32] Lumeng C.N., Bodzin J.L., Saltiel A.R. (2007). Obesity induces a phenotypic switch in adipose tissue macrophage polarization. J. Clin. Investig..

[33] Gao D., Madi M., Ding C., Fok M., Steele T., Ford C., Hunter L., Bing C. (2014). Interleukin-1β mediates macrophage-induced impairment of insulin signaling in human primary adipocytes. Am. J. Physiol.-Endocrinol. Metab..

[34] Myers M.G., Leibel R.L., Seeley R.J., Schwartz M.W. (2010). Obesity and leptin resistance: Distinguishing cause from effect. Trends Endocrinol. Metab..

[35] Friedman J.M. (2019). Leptin and the endocrine control of energy balance. Nat. Metab..

[36] Mancuso P. (2016). The role of adipokines in chronic inflammation. ImmunoTargets Ther..

[37] Trayhurn P. (2022). Adipokines: Inflammation and the pleiotropic role of white adipose tissue. Br. J. Nutr..

[38] Olefsky J.M., Glass C.K. (2010). Macrophages, inflammation, and insulin resistance. Annu. Rev. Physiol..

[39] Khanna D., Khanna S., Khanna P., Kahar P., Patel B.M. (2022). Obesity: A chronic low-grade inflammation and its markers. Cureus.

[40] Ye J. (2009). Emerging role of adipose tissue hypoxia in obesity and insulin resistance. Int. J. Obes..

[41] Shi H., Kokoeva M.V., Inouye K., Tzameli I., Yin H., Flier J.S. (2006). TLR4 links innate immunity and fatty acid–induced insulin resistance. J. Clin. Investig..

[42] Wen H., Gris D., Lei Y., Jha S., Zhang L., Huang M.T., Brickey W.J., Ting J.P. (2011). Fatty acid–induced NLRP3-ASC inflammasome activation interferes with insulin signaling. Nat. Immunol..

[43] Teng K.T., Chang C.Y., Chang L.F., Nesaretnam K. (2014). Modulation of obesity-induced inflammation by dietary fats: Mechanisms and clinical evidence. Nutr. J..

[44] Ravaut G., Légiot A., Bergeron K.F., Mounier C. (2020). Monounsaturated fatty acids in obesity-related inflammation. Int. J. Mol. Sci..

[45] Johnson G.H., Fritsche K. (2012). Effect of dietary linoleic acid on markers of inflammation in healthy persons: A systematic review of randomized controlled trials. J. Acad. Nutr. Diet..

[46] Rogero M.M., Calder P.C. (2018). Obesity, inflammation, Toll-like receptor 4 and fatty acids. Nutrients.

[47] Jiang C., Ting A.T., Seed B. (1998). PPAR-γ agonists inhibit production of monocyte inflammatory cytokines. Nature.

[48] Yessoufou A., Wahli W. (2010). Multifaceted roles of peroxisome proliferator-activated receptors (PPARs) at the cellular and whole organism levels. Swiss Med. Wkly..

[49] Cintra D.E., Ropelle E.R., Moraes J.C., Pauli J.R., Morari J., de Souza C.T., Grimaldi R., Stahl M., Carvalheira J.B., Saad M.J. (2012). Unsaturated fatty acids revert diet-induced hypothalamic inflammation in obesity. PLoS ONE.

[50] Yubero-Serrano E.M., Delgado-Lista J., Tierney A.C., Perez-Martinez P., Garcia-Rios A., Alcala-Diaz J.F., Castaño J.P., Tinahones F.J., Drevon C.A., Defoort C. (2015). Insulin resistance determines a differential response to changes in dietary fat modification on metabolic syndrome risk factors: The LIPGENE study. Am. J. Clin. Nutr..

[51] Schwingshackl L., Hoffmann G. (2014). Monounsaturated fatty acids, olive oil and health status: A systematic review and meta-analysis of cohort studies. Lipids Health Dis..

[52] Zhu L., Guo L., Xu J., Xiang Q., Tan Y., Tian F., Du X., Zhang S., Wen T., Liu L. (2024). Postprandial Triglyceride-Rich Lipoproteins-Induced Lysosomal Dysfunction and Impaired Autophagic Flux Contribute to Inflammation in White Adipocytes. J. Nutr..

[53] Muñoz A., Costa M. (2013). Nutritionally mediated oxidative stress and inflammation. Oxidative Med. Cell. Longev..

[54] Wang Y.I., Schulze J., Raymond N., Tomita T., Tam K., Simon S.I., Passerini A.G. (2011). Endothelial inflammation correlates with subject triglycerides and waist size after a high-fat meal. Am. J. Physiol.-Heart Circ. Physiol..

[55] Bai Y., Sun Q. (2015). Macrophage recruitment in obese adipose tissue. Obes. Rev..

[56] Clemente-Postigo M., Queipo-Ortuño M.I., Murri M., Boto-Ordoñez M., Pérez-Martínez P., Andres-Lacueva C., Cardona F., Tinahones F.J. (2012). Endotoxin increase after fat overload is related to postprandial hypertriglyceridemia in morbidly obese patients. J. Lipid Res..

[57] Laugerette F., Vors C., Alligier M., Pineau G., Drai J., Knibbe C., Morio B., Lambert-Porcheron S., Laville M., Vidal H. (2020). Postprandial endotoxin transporters LBP and sCD14 differ in obese vs. overweight and normal weight men during fat-rich meal digestion. Nutrients.

[58] Camargo A., Rangel-Zuñiga O.A., Haro C., Meza-Miranda E.R., Pena-Orihuela P., Meneses M.E., Marin C., Yubero-Serrano E.M., Perez-Martinez P., Delgado-Lista J. (2014). Olive oil phenolic compounds decrease the postprandial inflammatory response by reducing postprandial plasma lipopolysaccharide levels. Food Chem..

[59] Camargo A., Ruano J., Fernandez J.M., Parnell L.D., Jimenez A., Santos-Gonzalez M., Marin C., Perez-Martinez P., Uceda M., Lopez-Miranda J. (2010). Gene expression changes in mononuclear cells in patients with metabolic syndrome after acute intake of phenol-rich virgin olive oil. BMC Genom..

[60] Raz O., Steinvil A., Berliner S., Rosenzweig T., Justo D., Shapira I. (2013). The effect of two iso-caloric meals containing equal amounts of fats with a different fat composition on the inflammatory and metabolic markers in apparently healthy volunteers. J. Inflamm..

[61] Monfort-Pires M., Crisma A.R., Bordin S., Ferreira S.R. (2018). Greater expression of postprandial inflammatory genes in humans after intervention with saturated when compared to unsaturated fatty acids. Eur. J. Nutr..

[62] Bellido C., López-Miranda J., Blanco-Colio L.M., Pérez-Martínez P., Muriana F.J., Martín-Ventura J.L., Marín C., Gómez P., Fuentes F., Egido J. (2004). Butter and walnuts, but not olive oil, elicit postprandial activation of nuclear transcription factor κB in peripheral blood mononuclear cells from healthy men. Am. J. Clin. Nutr..

[63] Alayón A.N., Rivadeneira A.P., Herrera C., Guzmán H., Arellano D., Echeverri I. (2018). Metabolic and inflammatory postprandial effect of a highly saturated fat meal and its relationship to abdominal obesity. Biomedica.

[64] Emerson S.R., Kurti S.P., Harms C.A., Haub M.D., Melgarejo T., Logan C., Rosenkranz S.K. (2017). Magnitude and timing of the postprandial inflammatory response to a high-fat meal in healthy adults: A systematic review. Adv. Nutr..

[65] Cowan S., Gibson S., Sinclair A.J., Truby H., Dordevic A.L. (2022). Meals that differ in nutrient composition and inflammatory potential do not Differentially impact postprandial circulating cytokines in older adults above a healthy weight. Nutrients.

[66] Teeman C.S., Kurti S.P., Cull B.J., Emerson S.R., Haub M.D., Rosenkranz S.K. (2016). Postprandial lipemic and inflammatory responses to high-fat meals: A review of the roles of acute and chronic exercise. Nutr. Metab..

[67] O’Keefe J.H., Bell D.S.H. Postprandial Dysmetabolism: Understanding the Impact of Elevated Triglycerides After Meals. *Lipid Spin*, Spring Issue. 2020. https://www.lipid.org/lipid-spin/spring-2020/guest-editorial-postprandial-dysmetabolism-understanding-impact-elevated.

[68] Liddle D.M., Lin X., Ward E.M., Cox L.C., Wright A.J., Robinson L.E. (2021). Apple consumption reduces markers of postprandial inflammation following a high fat meal in overweight and obese adults: A randomized, crossover trial. Food Funct..

[69] Schwander F., Kopf-Bolanz K.A., Buri C., Portmann R., Egger L., Chollet M., McTernan P.G., Piya M.K., Gijs M.A., Vionnet N. (2014). A dose-response strategy reveals differences between normal-weight and obese men in their metabolic and inflammatory responses to a high-fat meal. J. Nutr..

[70] Klop B., Proctor S.D., Mamo J.C., Botham K.M., Castro Cabezas M. (2011). Understanding postprandial inflammation and its relationship to lifestyle behaviour and metabolic diseases. Int. J. Vasc. Med..

[71] Dubois V., Eeckhoute J., Lefebvre P., Staels B. (2017). Distinct but complementary contributions of PPAR isotypes to energy homeostasis. J. Clin. Investig..

[72] Chinetti G., Fruchart J.C., Staels B. (2000). Peroxisome proliferator-activated receptors (PPARs): Nuclear receptors at the crossroads between lipid metabolism and inflammation. Inflamm. Res..

[73] Hughes E., Wang X.X., Sabol L., Barton K., Hegde S., Myakala K., Krawczyk E., Rosenberg A.Z., Levi M. (2025). Role of Nuclear Receptors, Lipid Metabolism, and Mitochondrial Function in the Pathogenesis of Diabetic Kidney Disease. Am. J. Physiol.-Ren. Physiol..

[74] Saraf N., Sharma P.K., Mondal S.C., Garg V.K., Singh A.K. (2012). Role of PPARg2 transcription factor in thiazolidinedione-induced insulin sensitization. J. Pharm. Pharmacol..

[75] Zhang Y., Zhang X.Y., Shi S.R., Ma C.N., Lin Y.P., Song W.G., Guo S.D. (2024). Natural products in atherosclerosis therapy by targeting PPARs: A review focusing on lipid metabolism and inflammation. Front. Cardiovasc. Med..

[76] Cariou B., van Harmelen K., Duran-Sandoval D., van Dijk T.H., Grefhorst A., Abdelkarim M., Caron S., Torpier G., Fruchart J.C., Gonzalez F.J. (2006). The farnesoid X receptor modulates adiposity and peripheral insulin sensitivity in mice. J. Biol. Chem..

[77] Joseph S.B., Castrillo A., Laffitte B.A., Mangelsdorf D.J., Tontonoz P. (2003). Reciprocal regulation of inflammation and lipid metabolism by liver X receptors. Nat. Med..

[78] Hardie D.G., Ross F.A., Hawley S.A. (2012). AMPK: A nutrient and energy sensor that maintains energy homeostasis. Nat. Rev. Mol. Cell Biol..

[79] Ruderman N.B., Carling D., Prentki M., Cacicedo J.M. (2013). AMPK, insulin resistance, and the metabolic syndrome. J. Clin. Investig..

[80] Pourrajab B., Fotros D., Asghari P., Shidfar F. (2025). Effect of the Mediterranean Diet Supplemented with Olive Oil Versus the Low-Fat Diet on Serum Inflammatory and Endothelial Indexes Among Adults: A Systematic Review and Meta-analysis of Clinical Controlled Trials. Nutr. Rev..

[81] Itsiopoulos C., Mayr H.L., Thomas C.J. (2022). The anti-inflammatory effects of a Mediterranean diet: A review. Curr. Opin. Clin. Nutr. Metab. Care.

[82] Souza P.A., Marcadenti A., Portal V.L. (2017). Effects of olive oil phenolic compounds on inflammation in the prevention and treatment of coronary artery disease. Nutrients.

[83] Lian Z., Perrard X.Y.D., Peng X., Raya J.L., Hernandez A.A., Johnson C.G., Lagor W.R., Pownall H.J., Hoogeveen R.C. (2020). Replacing saturated fat with unsaturated fat in western diet reduces foamy monocytes and atherosclerosis in male Ldlr–/–mice. Arterioscler. Thromb. Vasc. Biol..

[84] Eichelmann F., Schwingshackl L., Fedirko V., Aleksandrova K.J. (2016). Effect of plant-based diets on obesity-related inflammatory profiles: A systematic review and meta-analysis of intervention trials. Obes. Rev..

[85] Poulsen N.B., Lambert M.N., Jeppesen P.B. (2020). The effect of plant derived bioactive compounds on inflammation: A systematic review and meta-analysis. Mol. Nutr. Food Res..

[86] Bolori P., Setaysh L., Rasaei N., Jarrahi F., saeid Yekaninejad M. (2019). Adherence to a healthy plant diet may reduce inflammatory factors in obese and overweight women-a cross-sectional study. Diabetes Metab. Syndr. Clin. Res. Rev..

[87] Escalante-Araiza F., Rivera-Monroy G., Loza-López C.E., Gutiérrez-Salmeán G. (2022). The effect of plant-based diets on meta-inflammation and associated cardiometabolic disorders: A review. Nutr. Rev..

[88] Fekete M., Lehoczki A., Kryczyk-Poprawa A., Zábó V., Varga J.T., Bálint M., Fazekas-Pongor V., Csípő T., Rząsa-Duran E., Varga P. (2025). Functional Foods in Modern Nutrition Science: Mechanisms, Evidence, and Public Health Implications. Nutrients.

[89] Jayarathne S., Koboziev I., Park O.H., Oldewage-Theron W., Shen C.L., Moustaid-Moussa N. (2017). Anti-inflammatory and anti-obesity properties of food bioactive components: Effects on adipose tissue. Prev. Nutr. Food Sci..

[90] Hirai S., Takahashi N., Goto T., Lin S., Uemura T., Yu R., Kawada T. (2010). Functional food targeting the regulation of obesity-induced inflammatory responses and pathologies. Mediat. Inflamm..

[91] Bartimoccia S., Cammisotto V., Nocella C., Del Ben M., D’Amico A., Castellani V., Baratta F., Pignatelli P., Loffredo L., Violi F. (2022). Extra virgin olive oil reduces gut permeability and metabolic endotoxemia in diabetic patients. Nutrients.

[92] He L., Su Z., Wang S. (2024). The anti-obesity effects of polyphenols: A comprehensive review of molecular mechanisms and signal pathways in regulating adipocytes. Front. Nutr..

[93] Deledda A., Annunziata G., Tenore G.C., Palmas V., Manzin A., Velluzzi F. (2021). Diet-derived antioxidants and their role in inflammation, obesity and gut microbiota modulation. Antioxidants.

[94] Mao T., Zhang Y., Kaushik R., Mohan M.S. (2024). Effects of polyphenols on gut microbiota and inflammatory markers in individuals with overweight or obesity: A systematic review and meta-analysis of randomized controlled trials. Crit. Rev. Food Sci. Nutr..

[95] Kiecolt-Glaser J.K., Belury M.A., Andridge R., Malarkey W.B., Glaser R. (2011). Omega-3 supplementation lowers inflammation and anxiety in medical students: A randomized controlled trial. Brain Behav. Immun..

[96] Li K., Huang T., Zheng J., Wu K., Li D. (2014). Effect of marine-derived n-3 polyunsaturated fatty acids on C-reactive protein, interleukin 6 and tumor necrosis factor α: A meta-analysis. PLoS ONE.

[97] Skulas-Ray A.C., Kris-Etherton P.M., Harris W.S., Vanden Heuvel J.P., Wagner P.R., West S.G. (2011). Dose-response effects of omega-3 fatty acids on triglycerides, inflammation, and endothelial function in healthy persons with moderate hypertriglyceridemia. Am. J. Clin. Nutr..

[98] Lyte J.M., Gabler N.K., Hollis J.H. (2016). Postprandial serum endotoxin in healthy humans is modulated by dietary fat in a randomized, controlled, cross-over study. Lipids Health Dis..

[99] McArdle M.A., Finucane O.M., Connaughton R.M., McMorrow A.M., Roche H.M. (2013). Mechanisms of obesity-induced inflammation and insulin resistance: Insights into the emerging role of nutritional strategies. Front. Endocrinol..

[100] Behall K.M., Scholfield D.J., Hallfrisch J.G., Liljeberg-Elmstahl H.G. (2006). Consumption of both resistant starch and β-glucan improves postprandial plasma glucose and insulin in women. Diabetes Care.

[101] Dewulf E.M., Cani P.D., Claus S.P., Fuentes S., Puylaert P.G., Neyrinck A.M., Bindels L.B., de Vos W.M., Gibson G.R., Thissen J.P. (2013). Insight into the prebiotic concept: Lessons from an exploratory, double blind intervention study with inulin-type fructans in obese women. Gut.

[102] Hall C.V., Hepsomali P., Dalile B., Scapozza L., Gurry T. (2024). Effects of a diverse prebiotic fibre blend on inflammation, the gut microbiota and affective symptoms in metabolic syndrome: A pilot open-label randomised controlled trial. Br. J. Nutr..

[103] Ríos-Covián D., Ruas-Madiedo P., Margolles A., Gueimonde M., De Los Reyes-gavilán C.G., Salazar N. (2016). Intestinal short chain fatty acids and their link with diet and human health. Front. Microbiol..

[104] Marco M.L., Heeney D., Binda S., Cifelli C.J., Cotter P.D., Foligné B., Gänzle M., Kort R., Pasin G., Pihlanto A. (2017). Health benefits of fermented foods: Microbiota and beyond. Curr. Opin. Biotechnol..

[105] Roager H.M., Licht T.R. (2018). Microbial tryptophan catabolites in health and disease. Nat. Commun..

[106] Krishnan S., Ding Y., Saedi N., Choi M., Sridharan G.V., Sherr D.H., Yarmush M.L., Alaniz R.C., Jayaraman A., Lee K. (2018). Gut microbiota-derived tryptophan metabolites modulate inflammatory response in hepatocytes and macrophages. Cell Rep..

